# Socio-economic impact of objectively-diagnosed allergy to staple foods in children and adolescents

**DOI:** 10.1186/2045-7022-5-S3-P14

**Published:** 2015-03-30

**Authors:** Jennifer Protudjer, Sven-Arne Jansson, Marianne Arnlind-Heibert, Ulf Bengtsson, Ann-Charlotte Sundqvist, Ingrid Kallström-Bengtsson, Birgitta Marklund, Roelinde Middelveld, Georgios Rentzos, Johanna Åkerström, Eva Östblom, Sven-Erik Dahlén, Staffan Ahlstedt

**Affiliations:** 1 The Centre for Allergy Research, Karolinska Institutet, Stockholm, Sweden; 2 Department of Medical Epidemiology and Biostatistics, Karolinska Institutet, Stockholm, Sweden; 3 Swedish Council on Health Technology Assessment, SBU, Stockholm, Sweden; 4 Department of Learning, Informatics, Management and Ethics, and Medical Management Centre, Karolinska Institutet, Stockholm, Sweden; 5 Allergy Unit, Sahlgrenska University Hospital, Gothenburg, Sweden; 6 Sachs' Children and Youth Hospital, Sodersjukhuset, Stockholm, Sweden; 7 The Swedish Asthma and Allergy Foundation, Stockholm, Sweden; 8 Department of Health and Caring Sciences, Linnaeus University, Kalmar, Sweden; 9 The Institute of Environmental Medicine, Karolinska Institutet, Stockholm, Sweden; 10 Department of Clinical Research and Education Sodersjukhuset, Karolinska Institutet, Stockholm, Sweden

## Background

Our group has previously described that indirect and intangible costs substantially burden households with a food allergic adult. We now extend our investigation to children and adolescents.

## Objective

To estimate the total, direct, indirect and intangible costs of food allergy, in households with at least one child or adolescent with objectively-diagnosed allergy to staple foods (cow's milk, wheat and/or hen's egg), and to compare these costs with age- and sex- matched controls.

## Methods

Participants included 84 children and 60 adolescents (cases), and 94 children and 56 adolescents (controls). Direct and indirect cost data collected via the Food Allergy Socio-Economic Questionnaire (developed by EuroPrevall) from parents of children and adolescents with objectively-diagnosed allergy to staple foods (cases) were compared to data from age- and sex-matched controls, and calculated as annual household costs. Total costs were defined as direct + indirect costs. Direct and indirect costs were also considered independently. Intangible costs included measures of self-reported health, standard of living and losses of well-being.

## Results

Annual total household costs were significantly higher for cases than controls, for children (20,808€ vs. 16,850€, p<0.05) and adolescents (23,456 € vs. 18,666 €), and were driven by direct (e.g. medicines) and indirect (e.g. time spent with health professionals) costs. Children, but not adolescents, with a history of anaphylaxis had higher annual direct costs vs. those without anaphylaxis (13,016€ vs. 10,044€, p<0.05). Intangible costs were greater amongst cases than controls for both age groups (e.g. self-reported health p<0.01).

## Conclusion

Households with children and adolescents with objectively-diagnosed allergy to staple foods have higher total household costs than controls. Direct and indirect household costs were significantly higher for food allergic children, but not food allergic adolescents, compared to controls. Among both children and adolescents, objectively-diagnosed allergy to staple foods adversely impacts intangible costs.

